# Complete Genome Sequence of a Swiss Hepatitis E Virus Isolate from the Liver of a Fattening Pig

**DOI:** 10.1128/genomeA.00113-18

**Published:** 2018-03-01

**Authors:** Valerie Wist, Jakub Kubacki, Julia Lechmann, Marco Steck, Cornel Fraefel, Roger Stephan, Claudia Bachofen

**Affiliations:** a Institute of Virology, Vetsuisse Faculty, University of Zurich, Zurich, Switzerland; b Institute for Food Safety and Hygiene, Vetsuisse Faculty, University of Zurich, Zurich, Switzerland

## Abstract

We present here the full-length genome sequence of a hepatitis E genotype 3 virus (HEV-3) isolate, CH_VW117, from the liver of a healthy fattening pig collected at the slaughter level. Sequence analysis implies that this strain belongs to the newly proposed HEV subtype 3s.

## GENOME ANNOUNCEMENT

Hepatitis E virus (HEV) is a small non- or quasienveloped virus (1) with a positive-sense single-stranded RNA genome of approximately 7.2 kb, and it belongs to the family Hepeviridae. HEV genotypes 1 and 2 (HEV-1 and HEV-2, respectively) circulate within the human population and are a major health issue in developing countries. HEV-3 and HEV-4 are zoonotic viruses that are highly prevalent in porcine species and may be transmitted to humans by the consumption of pig liver and meat (2). Recently, the full-length genome sequences of two HEV-3 isolates (from a human stool sample and a raw sausage containing pig liver) of the first confirmed Swiss case of a foodborne hepatitis E virus infection were reported (3).

Here, we present the full-length genome sequence of an HEV isolate, CH_VW117, from the liver of a fattening pig collected at the abattoir level. The animal originated from the northern part of Switzerland and was slaughtered in November 2017. Total RNA was extracted using the QIAamp viral RNA minikit, according to the manufacturer’s instructions (Qiagen GmbH, Germany). The RNA was shown to be HEV positive by a commercial quantitative real-time reverse transcription-PCR (RT-PCR) (Ceeramtools; bioMérieux, Geneva, Switzerland). To prepare the RNA samples for next-generation sequencing (NGS), sequence-independent single-primer amplification was performed (4), and the purified amplicons were used for the construction of sequencing libraries using the NEBNext Ultra II library preparation kit (BioConcept, Allschwil, Switzerland). A paired-end NGS run of 2 × 150-nucleotide read length was performed at the Functional Genomic Center Zurich using the Illumina NextSeq500 machine. Alignment of the reads to full-length hepatitis E virus genomes using SeqMan NGen software (DNASTAR Lasergene, Madison, WI, USA) revealed the best matches (100% identity) to be the recently published Swiss HEV-3 strains MF346772 and MF346773 (3). The new isolate adds more evidence to the proposed existence of a Switzerland-specific HEV-3 subcluster, listed as subcluster 3s, in the public Hepatitis E Virus Genotyping Tool of HEVnet (http://www.rivm.nl/mpf/typingtool/hev/).

### Accession number(s).

This sequence is deposited in GenBank under the accession number MG573193.
